# Flexible ureteroscopy versus miniaturized percutaneous nephrolithotomy for renal stones of 1–2 cm.

**DOI:** 10.12703/r/9-29

**Published:** 2020-12-22

**Authors:** M Hammad Ather, M Nasir Sulaiman

**Affiliations:** 1 Department of Surgery, Aga Khan University, Karachi, Pakistan

**Keywords:** Mini perc, micro perc, ultra mini perc, super mini perc, RIRS, Flexible URS, miniaturized PCNL, renal stones

## Abstract

Technological advances and innovation in endourology have significantly reduced the indications of extracorporeal shockwave lithotripsy in the management of moderate-sized renal stones. In the last decade, we have witnessed a trend towards the use of finer scopes for percutaneous procedures instead of standard percutaneous nephrolithotomy (PCNL) (≥22 Fr). Miniaturized PCNL (mPCNL), i.e. miniPCNL (12–20 Fr), ultra-miniPCNL (11–13 Fr), mini-microPCNL (8 Fr), and microPCNL (<5 Fr), is increasingly being used. Concomitant developments in laser technology have provided a safe and effective stone fragmentation modality for use via flexible ureteroscopes (fURS). Technological advances in the design of fURS have improved not only the optics (fiber optic to chip-on-the-tip technology digital image) but also the ergonomics. Both the endourological techniques are extremely effective and safe, as shown in a multitude of good-quality studies. There are some differences in stone-free rate and complications. mPCNL in general has a higher stone-free rate, albeit with a slightly higher incidence of hemorrhagic complications. fURS often requires longer stenting time and longer period to achieve stone clearance, whereas mPCNL often needs ureteral catheter for only 24 hours and has a higher first day stone-free rate. fURS is a 1 day procedure compared to mPCNL, which requires patients to stay hospitalized for 2–3 days. It is therefore important to tailor the indications of these two procedures to the individual patient’s needs.

## Introduction

Progress in the field of endourology has superseded technological advances in extracorporeal shockwave lithotripsy (ESWL). Whereas ESWL was the mainstay of treatment for most non-lower caliceal renal stones, it is now relegated to a lower order of preference. ESWL ruled the stone world from its introduction in the mid-1980s^1^ until about early 2000, when flexible ureteroscopy (fURS) and miniaturized percutaneous nephrolithotomy (mPCNL) took over as the mainstay of treatment for most 10–20 mm renal stones.

The aims of treatment for 10–20 mm renal stones include achieving stone-free status, preferably in a single session, with a low complication rate and without the need for ancillary interventions. For the small stones, it is preferable to avoid using stents and nephrostomy tubes as well.

The need for intervention for moderate-sized renal stones is often due to symptoms. They are also often associated with recurrent infections and, rarely, stone growth and obstruction. The natural history of small and medium stones is variable. Stone growth and symptomatic events are often seen in patients with competing morbidities like diabetes and hyperuricemia^2^ in adult urolithiasis. Sheth and colleagues^3^ recently made similar observations in pediatric patients with small kidney stones.

## Parameters for comparison

The choice between fURS and mPCNL is an area of current active research. The two procedures are compared for safety and efficacy in many contemporary randomized and non-randomized studies. The efficacy is assessed in terms of stone-free rate immediately following the procedure and at 1 and 3 months afterwards. The need for ancillary and repeat procedures can be assessed by efficiency quotient^4,5^. The safety assessment of the two procedures can be done by a generic standardized tool like Clavien-Dindo scale or by a specialized tool for fURS, i.e. post ureteroscopy lesion scale (PULS)^6^. In addition, cost effectiveness^7^ and patient satisfaction^8^ are other important factors to consider when comparing the two modalities.

## How to select the right procedure

The contemporary surgical practice involves shared decision making between surgeon and patient. Omar *et al*.^9^, in work reported a few years ago, evaluated the factors that impact patients’ preferences on choosing ESWL or URS for the management of an asymptomatic stone. They noted that patients’ preferences mainly rely on physicians’ recommendations. In order to assist patients in making a shared decision, Gökce *et al*.^10^ developed a decision aid for symptomatic non-lower pole renal stones <20 mm in size. The authors noted that the decision aid made a positive impact on patients’ level of knowledge on stone disease and the particular treatment options.

## Stent use in the two procedures

In a recently reported systematic review and meta-analysis^11^ comparing microPCNL and fURS in the management of moderately sized kidney stones, Zhang and colleagues noted that percutaneous procedures are associated with lesser need for double J stents and higher stone-free rate but at the cost of greater drop in hemoglobin and longer hospital stay.

Double J stents are related to significant bother in patients undergoing stone surgery^12^. Many techniques in the placement, stent material, and post placement medical treatments have been described to ameliorate the stent-related symptoms. However, there is no single treatment that works for all patients. Anti reflux stents^13^, placing the distal end away from the trigone, and using silicone rather than polytetrafluoroethylene stents are some of the ways of improving stent-related morbidity. Various medical options including anti-cholinergic drugs and alpha-blockers have been explored in clinical trials^14^.

The placement of a double J stent is part of most fURS and mPCNL procedures for stones. It is needed prior to the procedure to facilitate and ease the passage of the access sheath and post procedure to facilitate the passage of fragments. Similarly, most mPCNL procedures without external drainage use double J stents. There are reports indicating that the access sheath can safely be placed in unstented ureters^15^. However, this often results in the placement of a smaller access sheath, which does not provide enough space between the scope and inner access sheath to allow free drainage of fluid. In view of the use of small caliber nephroscopes, ultra-miniPCNL, super-miniPCNL (SMP), and microPCNL use laser as standard stone fragmentation energy^16^. However, the majority of these procedures uses a tubeless technique. Most mPCNL employs some form of suction and drainage to allow the passage of most of the dust and most significant fragments. The development of miniaturized scopes facilitated knowledge of the physics behind the vacuum cleaner effect generated during procedures^17^.

## Stone location

Lower pole stones provide unique difficulty in stone clearance^18^. ESWL is the least effective and mPCNL is the most effective, with fURS outcomes in between. Kandemir *et al*.^19^ in a prospective randomized study compared microPCNL with fURS for <15 mm lower caliceal stones. They noted no difference in the stone-free rate (*P* = 0.158), operating time, pre-operative–post-operative hemoglobin, serum creatinine, and estimated glomerular filtration rate (eGFR) values. ESWL failure in stone clearance for lower caliceal stone is attributed to unique anatomical parameters^20^. Recently, Karim *et al*.^21^ revisited the lower pole caliceal anatomy *viz a viz* infundibular pelvic angle, infundibular length, and infundibular width in a systematic review. The authors noted that the stone-free rate ranged from 78 to 88%, and infundibular pelvic angle was found to be the most important predictor of treatment outcomes. The other significant factors impacting stone-free rate include stone size and hardness. Jiao *et al*.^22^ recently analyzed the safety and efficacy of both minimally invasive PCNL and fURS in a systematic review. They noted that mPCNL is more effective in the treatment of renal stones compared to fURS, particularly in the lower pole calyx between 10 and 20 mm. However, they noted that mPCNL is associated with a longer hospital stay and a higher incidence of hematoma formation.

## Different types of mPCNL

Comparing SMP and fURS, Zeng *et al*.^23^ noted that a higher stone-free rate using ultrasound and plain X-ray of the kidneys, ureters, and bladder (KUB) on day 1 after surgery was in favor of SMP (91.2% vs. 71.2%); however, CT at 3 months narrowed the difference to 93.8% vs. 82.5% for SMP and fURS, respectively. Comparing safety profile, the authors noted hemoglobin drop and pain score were higher for SMP compared to fURS; however, there was no need for transfusion in either group. In a meta-analysis comparing PCNL using various Amplatz sheath sizes with fURS, Gao *et al*.^24^ analyzed 14 publications and 1,279 patients. They noted that overall stone-free rate and location in the lower pole calyx were statistically significantly different between PCNL and retrograde intrarenal surgery (RIRS), favoring PCNL. However, again, safety favors fURS, which was associated with shorter hospital stay (*P* = 0.0001) and less blood loss (*P* = 0.00001).

## Cost impact

fURS before the introduction of disposable scopes was considered an expensive modality. The equipment breakdown rate of 5.34% with 21 major incidents in a period of 4 years^25^ was noted at a university hospital. The cost comparison, which is globally acceptable between the two procedures, is often difficult. In a cost comparison between fURS and microPCNL, Bagcioglu *et al*.^26^ noted the mean cost of RIRS was $917.13 ± 73.62 and the mean cost of mPCNL was $831.58 ± 79.51; this difference was statistically significant (*P* <0.001). Pan and colleagues^27^, however, in a Chinese medical setting noted that there was no difference in the overall cost of fURS and mPCNL in a non-randomized comparison for 2–3 cm kidney stones. In general, fURS single procedure is costlier than mPCNL. The use of disposable items like baskets, access sheaths, and disposable flexible ureteroscopes incurs an additional cost. Besides this capital equipment cost of laser and reusable flexible scopes, frequent breakdowns and maintenance add to the overall cost to the healthcare system. The scopes used for mPCNL are almost all reusable solid steel alloy with long life; the use of disposables is limited. Determination of cost effectiveness analysis (CEA) is again highly dependent on the healthcare system, insurance, and the trade-offs between the costs and health effects of the two interventions for moderate-sized renal stones. The resultant metrics facilitate informed decisions in introducing or continuing an intervention. Effectiveness outcomes from CEA of fURS and mPCNL are assessed by prevention of stone recurrence and quality-adjusted life-year (QALY)^28^. Other important considerations for interventional stone management include efficiency quotient rather than stone-free rate, return to work, and minimal long-term complications.

## Stones in children

In the pediatric population, studies have shown similar outcomes to those conducted in adults. Chen *et al*.^29^ analyzed 11 studies, which included one randomized controlled trial, four retrospective case-control studies, and six case series with a total of 822 children. They noted that significantly shorter hospital stay and fluoroscopy time were needed for RIRS than for PCNL. They also noted that the overall complications were higher for PCNL compared with RIRS (odds ratio 1.70, 95% confidence interval 1.02–2.84; *P* = 0.04). However, no significant differences were found in initial and final stone-free rate and operative times (*P* >0.05). In a single-arm observational study, Sofimajidpour *et al*.^30^, while treating children younger than 8 years old with 1–2 cm kidney stones, noted that ultra-miniPCNL is safe, with a stone-free rate of over 95%. However, unlike those of adults, pediatric ureters are much more efficient in clearing kidney stones following ESWL^31^. The 1–2 cm renal stones can be managed safely and efficiently by lithotripsy. Informed decision making for 1–2 cm pediatric kidney stones should include ESWL besides mPCNL and fURS. High-density stones (Hounsfield Unit>1,000), close to 20 mm lower pole stones, and stones refractory to ESWL are clear indications for interventional endourological treatment. In a randomized prospective study comparing mPCNL and ESWL in pediatric kidney stones, Farouk *et al*. noted that the stone-free rate is comparable in the two groups; however, the ESWL had a higher re-treatment rate, and mPCNL was associated with more radiation exposure and need for in-patient stay. They noted no difference in morbidity in the two groups.

## Conclusions

In conclusion, both modalities are comparable in terms of overall safety and efficacy. fURS has a better safety profile and mPCNL is more efficacious. With improvement in technique and equipment, routine use of pre-stenting in fURS is no longer encouraged. It is important that treatment options be tailored to the needs of the individual patient, availability of equipment, and local expertise. In children, ESWL should be considered as a valid option for most moderate-sized kidney stones.
